# Polysaccharopeptides derived from *Coriolus versicolor *potentiate the S-phase specific cytotoxicity of Camptothecin (CPT) on human leukemia HL-60 cells

**DOI:** 10.1186/1749-8546-5-16

**Published:** 2010-04-27

**Authors:** Jennifer Man-Fan Wan, Wai-Hung Sit, Xiaotong Yang, Pingping Jiang, Leo Lap-Yan Wong

**Affiliations:** 1 Agricultural, Food and Nutritional Sciences Division, School of Biological Sciences, The University of Hong Kong, Pokfulam Road, Hong Kong SAR, China; 2 Institutes of Microbiology and Immunology, Shanghai Normal University, 100 Guilin Road, Shanghai 200234, China

## Abstract

**Background:**

Polysaccharopeptide (PSP) from *Coriolus versicolor *(*Yunzhi*) is used as a supplementary cancer treatment in Asia. The present study aims to investigate whether PSP pre-treatment can increase the response of the human leukemia HL-60 cells to apoptosis induction by Camptothecin (CPT).

**Methods:**

We used bivariate bromodeoxyuridine/propidium iodide (BrdUrd/PI) flow cytometry analysis to measure the relative movement (RM) of the BrdUrd positively labeled cells and DNA synthesis time (Ts) on the HL-60 cell line. We used annexin V/PI flow cytometry analysis to quantify the viable, necrotic and apoptotic cells. The expression of cyclin E and cyclin B1 was determined with annexin V/PI flow cytometry and western blotting. Human peripheral blood mononuclear cells were used to test the cytotoxicity of PSP and CPT.

**Results:**

PSP reduced cellular proliferation; inhibited cells progression through both S and G_2 _phase, reduced ^3^H-thymidine uptake and prolonged DNA synthesis time (Ts) in HL-60 cells. PSP-pretreated cells enhanced the cytotoxicity of CPT. The sensitivity of cells to the cytotoxic effects of CPT was seen to be the highest in the S-phase and to a small extent of the G_2 _phase of the cell cycle. On the other hand, no cell death (measured by annexin V/PI) was evident with the normal human peripheral blood mononuclear cells with treatment of either PSP or CPT.

**Conclusion:**

The present study shows that PSP increases the sensitization of the HL-60 cells to undergo effective apoptotic cell death induced by CPT. The pattern of sensitivity of cancer cells is similar to that of HL-60 cells. PSP rapidly arrests and/or kills cells in S-phase and did not interfere with the anticancer action of CPT. PSP is a potential adjuvant to treat human leukemia as rapidly proliferating tumors is characterized by a high proportion of S-phase cells.

## Background

The polysaccharopeptide (PSP) extract of the Chinese medicinal mushroom *Coriolus versicolor *(*Yunzhi*) has been used in Asia to treat cancer as well as to alleviate weakness, improve immunological activities, appetite and comfort for patients [1]. The strong immunomodulatory effects of PSP such as elevation of IL-2, natural killer cell activity and T-cell proliferation may be beneficial to advanced cancer patients with depressed immunity [2,3]. PSP can be potentially used to treat cancer due to its ability to distinguish cancerous cells from normal cells [4-6]. However, concerns remain that the PSP extract may diminish the effectiveness of chemotherapeutic regimens because little is known about the PSP anti-tumor action mechanism. Our recent studies showed that instead of interfering with the anticancer actions of drugs PSP potentiated the anticancer effects of doxorubicin and etoposide in human leukemia cells [3] and human breast cancer cells through interfering the S phase progression and DNA synthesis [4]. As PSP synchronizes cells in S-phase, we hypothesize in this study that PSP may serve as a chemotherapeutic agent in combination therapy with other S-phase targeted drugs such as Camptothecin (CPT). CPT is a cytotoxic quinoline alkaloid isolated from the bark and stem of *Camptotheca acuminate *(*Camptotheca*, *Xishu*, Happy Tree), a native plant of China. CPT kills cancer cells by interfering with the function of DNA topoisomerase I (TopI) during DNA replication in the S-phase [7,8]. Incubation of cells with CPT stabilizes the TopI-DNA cleavable complexes, leading to fragmentation of nuclear DNA [8]. The cell sensitivity to cytotoxic effects of CPT was reported to reach the highest in the late S-phase and early G_2 _[9]. CPT demonstrated remarkable anticancer activity in preliminary clinical trials but also produced side effects such as leucopenia [10].

Using the S-phase synchronization strategy, the present study tests the hypothesis that pre-exposing the human myelogenous leukemia HL-60 cells to PSP increases cell death at low concentrations.

## Methods

### Preparation of PSP extract

PSP (340 mg/capsule, Winsor Health Products Ltd, Hong Kong) powder was dissolved in distilled water by boiling. All water soluble fractions were collected, centrifuged and freeze-dried for a dry power. PSP stock solutions of 10 mg/ml were prepared from the dry powder to be used in all experiments.

### Cell line and cell culture

HL-60 cells were routinely cultured in RPMI 1640 medium supplemented with 10% fetal bovine serum. Cells were cultured at 37°C in a humidified atmosphere with 5% CO_2_. The cultures were supplied with fresh complete medium and cell density was adjusted to 1 × 10^5 ^cells/ml per flask every three days to maintain asynchronicity and exponential growth. Cell densities were monitored to ensure that they did not exceed 1 × 10^6 ^cells/ml. In all experiments, cells were fed with fresh complete medium a day before experiments. Human peripheral blood (Buffy coat) obtained from healthy donors (age ranged 20-40 years) was provided by the Hong Kong Red Cross Blood Transfusion Service. Human peripheral blood mononuclear cells (PBMCs) were isolated with Ficoll-Paque density gradient centrifugation (Pharmacia Biotech, Sweden). Briefly, the diluted Buffy coat (1:1 with RPMI medium) was layered on the Ficoll-Paque and centrifuged at 400 g for 30 minutes at room temperature. The PBMCs were washed twice with RPMI medium and then suspended in the RPMI medium.

A series of dilutions was prepared from the PSP stock solution (10 mg/ml) in RPMI and incubated with the HL-60 cells for 24, 48 and 72 hours, respectively. For combination treatment of PSP and CPT, the HL-60 cells were first exposed to PSP (25 μg/ml) for 72 hours before being exposed to CPT (1 μM) for five more hours. The PBMCs were treated with PSP and CPT in the same manner.

### Cell proliferation and [^3^H] thymidine uptake assay

Cell proliferation and cell size were determined every 24 hours with Beckman Coulter Multisizer (Beckman Coulter Electronics Ltd, USA). Cell suspension was diluted in 20 ml PBS and the mixture was analyzed with the multisizer. Cell viability was evaluated with the Trypan Blue Exclusion test (Sigma, USA).

For [^3^H] thymidine uptake assay, HL-60 cells (1 × 10^4 ^per well) were pipetted onto a 96-well plate. After incubation with or without PSP, the cells were pulsed with 0.5 μCi/well of [^3^H] thymidine for six hours before harvest. Incorporated radioactivity was counted in a liquid scintillation counter (Beckman LS3000, Beckman, USA). Each concentration was tested in quadruplicates and repeated on three separate occasions.

### BrdUrd/PI immunostaining analysis by flow cytometry

Bromodeoxuridine (BrdUrd) propidium iodide (PI) flow cytometry was used to determine DNA synthesis time (Ts) via measurement of the relative movement of the BrdUrd labeled S-phase cells as previously described [11-13]. Cells were pulsed with BrdUrd (10 μM) in the last 20 minutes of PSP incubation and were washed, re-suspended in warm complete culture medium with or without PSP and further incubated for one and six hours for BrdUrd analysis. After incubation, the cells were washed with PBS and fixed with ice-cold ethanol (75%w/v in Milli-Q water) and the fixed samples were stored at -20°C for further analysis. Fixative was removed by centrifugation and the cell pellets were washed with PBS. DNA was partially denatured by incubation with 1 M HCl for 20 minutes at room temperature, after which the cells were washed three times with PBS containing 0.05% Tween-20. Cells were incubated with 100 μl of anti-BrdUrd monoclonal antibody (5 μg/ml; 1:100) for one hour and further incubation with a FITC-conjugated goat anti-mouse IgG antibody (100 μl; 1:40) in the dark for another 30 minutes. The cells were stained with PI staining solution (50 μg/ml PI, 10 μg/ml RNase, 0.01 M Tris and 10 mM NaCl) before flow cytometric analysis. The amount of DNA distribution in the difference phases of the cell cycle was analyzed with the use of the WinList (Version 1.04, Verity Software House Inc., USA) and ModFit software (Version 5.11, Verity Software House Inc., USA).

Relative movement (RM) and DNA synthesis time (Ts) were calculated as previously described [6,11-15]:

where F_lud(t)_, F_G0/G1 _and F_G2/M _are the mean PI fluorescence (DNA content) of the BrdUrd labeled undivided population G_0_/G_1 _and G_2_/M at time t (hour) after BrdUrd pulsing respectively. RM(0) and RM(t) are the relative movement values at time 0 and t (hour) respectively after BrdUrd pulsing.

### Quantification of apoptosis by annexin V/PI flow cytometry

Using the annexin V binding assay with the Apoptosis Detection Kit (Trevigen Inc., USA) and flow cytometry, we determined the apoptosis induction effect of CPT with and without PSP treatment on both HL-60 cells and human peripheral blood mononuclear cells. Cells (2 × 10^5^) after incubation with or without PSP and CPT were harvested and centrifuged at 400 × g for five minutes to remove the culture medium. Cell pellets were washed with 3 ml PBS and re-suspended in 500 μl binding buffer. After centrifugation and removal of the binding buffer, 100 μl of annexin V incubation reagent (10 μl 10 × binding buffer, 10 μl propidium iodide, 1 μl annexin V conjugate and 79 μl Milli-Q water) were added to each sample. Samples were incubated for 15 minutes in the dark at room temperature. Cell suspension was then diluted with 400 μl binding buffer and was analyzed with a Coulter's Epic Elite flow cytometer (Beckman and Coulter Electronics Ltd, USA).

### Bivariate analysis of cyclin E and cyclin B_1 _expression by flow cytometry

Cells were harvested into 15 ml centrifugation tubes. All samples were washed twice with 10 ml PBS and centrifuged at 400 g for five minutes. Supernatant was removed and cell pellets were fixed with ice-cold ethanol (75%w/v in Milli-Q water). The fixed samples were stored at -20°C for further analysis.

The fixed samples were washed with 10 ml PBS-BSA (1%). The cells were incubated with 100 μl of anti-cyclin E monoclonal antibody (5 μg/ml; 1:100) or anti-cyclin B_1 _monoclonal antibody (1 μg/ml; 1:100) for one hour. Mouse monoclonal isotype controls at equivalent concentration were used instead of the anti-cyclin antibodies for the background fluorescence check. After incubation, the primary antibodies of the cells were washed twice with PBS-BSA, labeled with FITC-conjugated goat anti-mouse IgG antibody (100 μl; 1:40) and kept in the dark at room temperature for one hour. The cells were stained with PI solution before flow cytometric analysis.

### Western blot analysis of the expression of cyclin E and cyclin B_1_

Cells were harvested and washed twice with ice-cold PBS (45 ml) followed by centrifugation at 400 × g for five minutes. Cell pellet was re-suspended in lysis buffer (5 × 10^6 ^cells/100 μl) with HEPES (25 mM; pH7.5), NaCl (150 mM), EDTANa_2 _(1 mM), DTT (1 mM), Triton × 100 (1%) and protease inhibitor cocktail. The suspension was then frozen and thawed three times with cold methanol at -80°C before it was placed in ice for further 30 minutes and then centrifuged. After centrifuged at 14,000 × g for 30 minutes at 4°C, the suspension was collected and stored at -80°C. Protein quantity was determined with Bradford assay. Protein extracts were mixed with an equal volume of 2× sample buffer (0.125 M Tris-HCl, 4% SDS, 20% v/v glycerol, 0.2 M DTT, 0.02% bromophenol blue, pH6.8) and the mixture was heated in boiling water for three minutes.

Equal amounts of total protein (20 μg) were subjected to 12.5% SDS-PAGE followed by Western blotting onto a PVDF membrane (GE Healthcare Life Sciences, USA). Membranes were incubated with anti-cyclin E antibodies and detected with the matching species specific secondary HRP-conjugated antibodies. Proteins were detected with the ECL system (GE Healthcare Life Sciences, USA) and the band intensity was measured with Quantity One software (Bio-Rad, USA).

### Statistical analysis

All data are presented as mean standard deviation (SD). Statistical differences between the treatment and control groups were determined by post-hoc Duncan's test with SPSS statistical software for Windows, version 12·0 (SPSS Inc., USA). Probability values (P) < 0.05 were considered statistically significant.

## Results

### PSP reduced HL-60 cells proliferation and [^3^H] thymidine uptake

**Figure 1:**
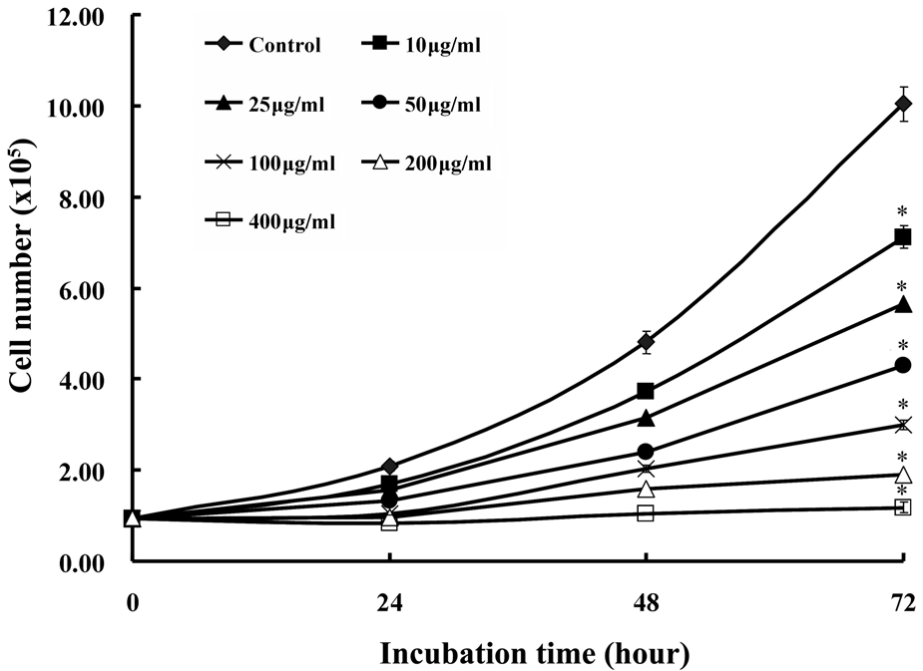
**The dose and time effect of PSP on human leukemia HL-60 cell proliferation**. HL-60 cells were treated with various PSP concentrations (0-400 μg/ml) for 24, 48 and 72 hours. Cells proliferation was determined with cell densities analysis on the Multisizer. Values are expressed as mean (SD) (*n *= 4). * *P *< 0.001, vs. control.

**Figure 2:**
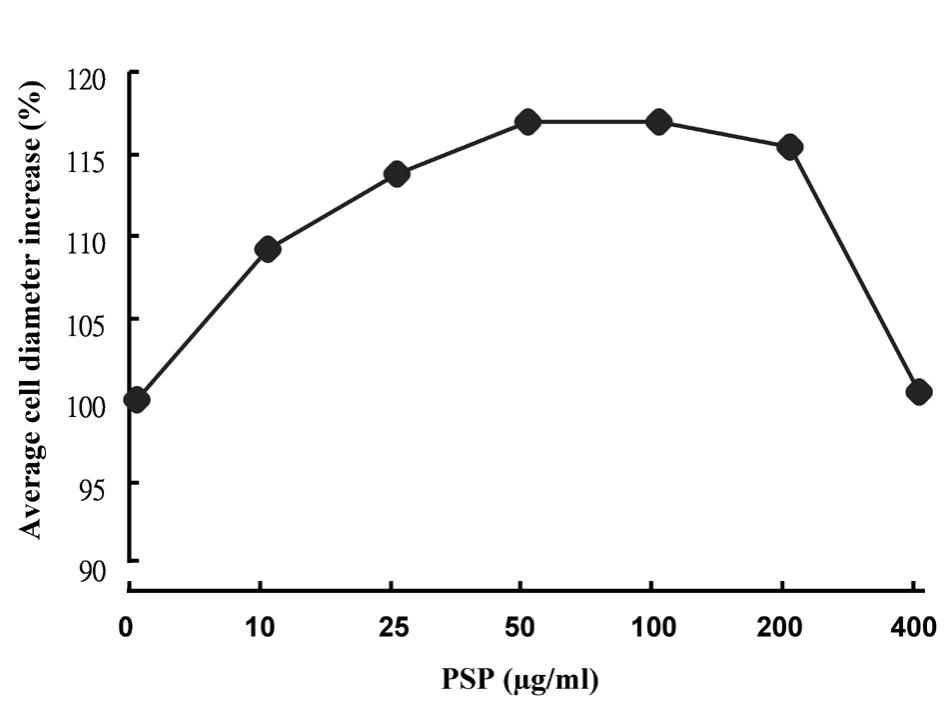
**Effect of PSP on the average size of HL-60 cells**. HL-60 cells were treated with various PSP concentrations (0-400 μg/ml) for 72 hours. Average diameter of HL-60 cells was measured with the Multisizer.

**Figure 3:**
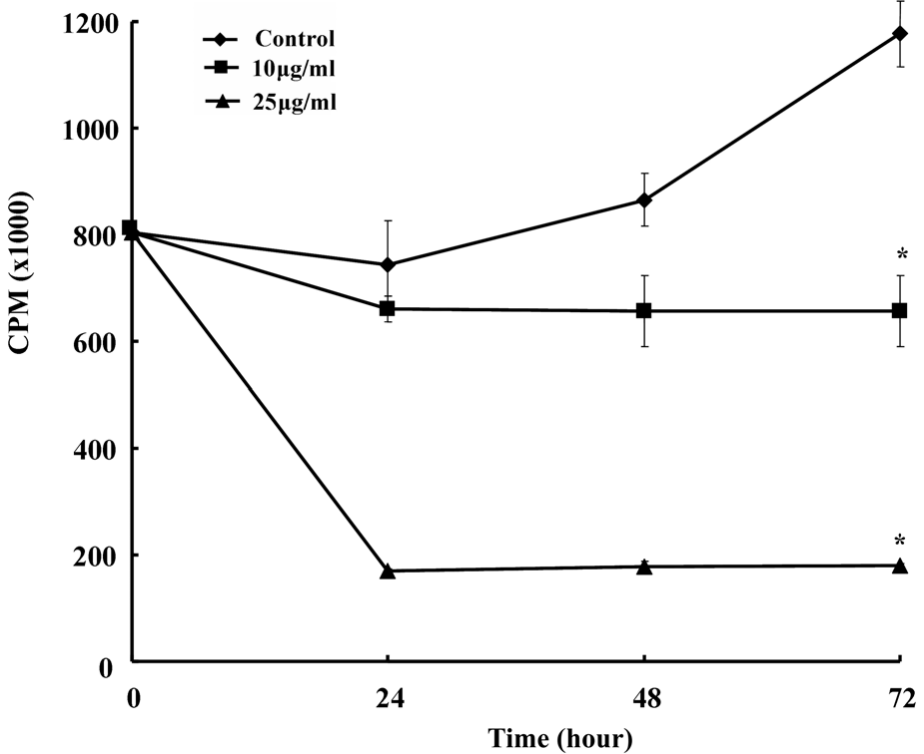
**Effect of PSP on [^3^H] thymidine uptake by HL-60 cells**. Cells were pulsed with [^3^H] thymidine (0.5 Ci/well) for six hours after treatment with or without PSP. Cells were harvested and counted at 24 hour intervals. Values are expressed as mean (SD) (*n *= 4). * *P *< 0.001, vs. control.

PSP reduced HL-60 cells proliferation in a dose- and time-dependant manner (Figure 1). The average cell size of the HL-60 cells increased as PSP doses increased. The mean diameter of the cells increased 13.82% after 72 hours of PSP treatment at 25 μg/ml (Figure 2). The cell size increase may be due to: (1) increased DNA content, (2) increased protein synthesis, (3) inhibition of cells division or (4) all of these events. The [^3^H] thymidine incorporation in the HL-60 cells was reduced by 77.2%, 79.4% and 84.7% for treatment times of 24, 48 and 72 hours respectively (Figure 3), suggesting DNA synthesis was reduced in cells exposed to PSP.

### Cell cycle arrest induced by PSP in S and G_2_/M phase

**Figure 4:**
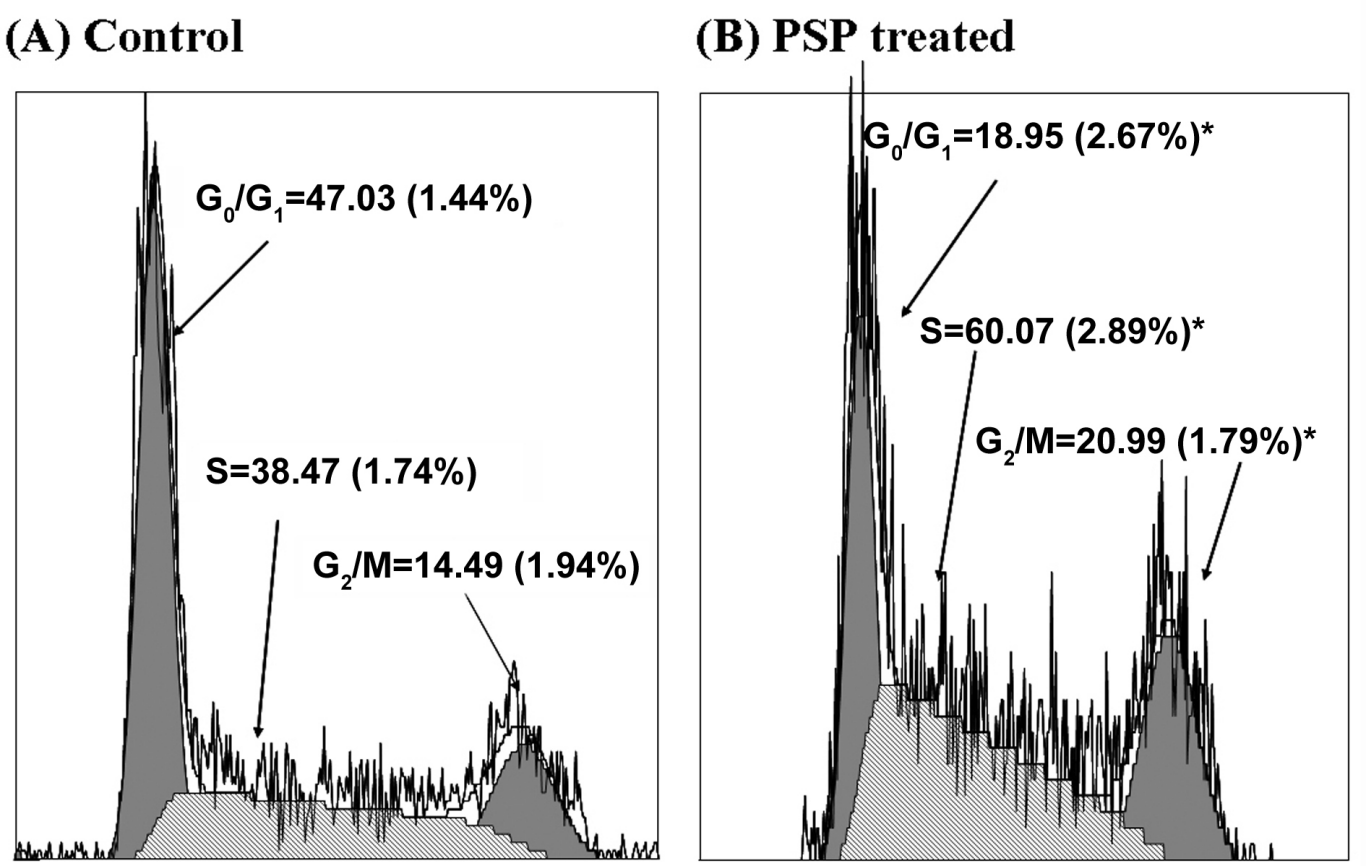
**Effect of PSP on HL-60 cell cycle distribution**. HL-60 cells were treated with PSP (25 μg/ml) for 72 hours. Cell cycle distribution in G_0_/G_1_, S and G_2_/M phases was measured by DNA/PI flow cytometry. Values are expressed as mean (SD) (*n *= 5). * *P *< 0.001, vs. control.

**Figure 5:**
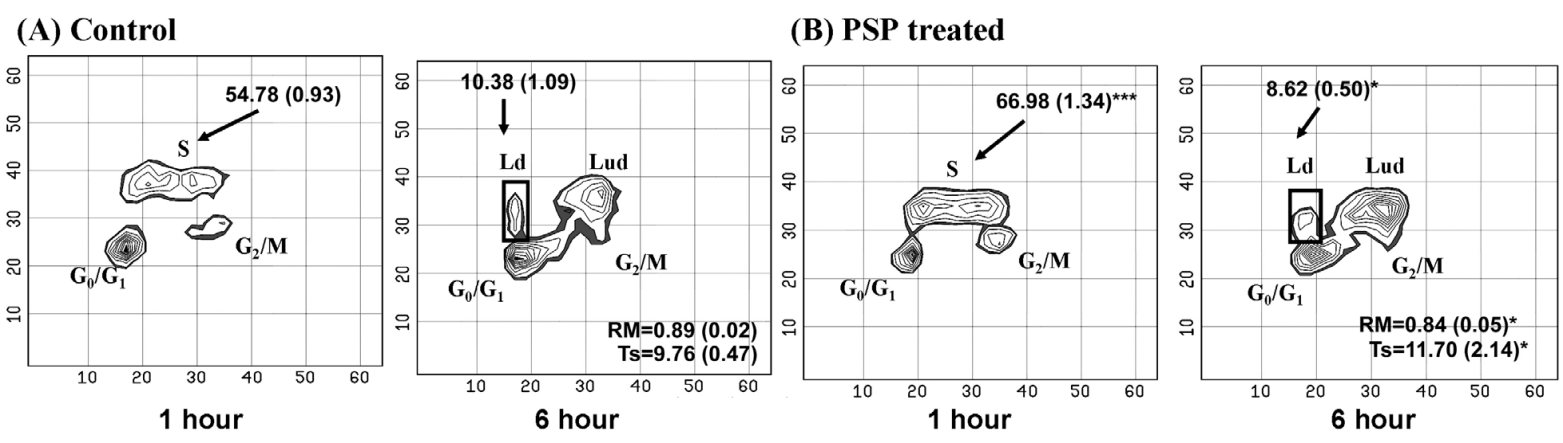
**Bivariate BrdUrd/PI labeling of DNA with flow cytometric analysis**. Contour plots showing cells with and without BrdUrd labeling after one hour and six hours of BrdUrd incubation. Cells were pulsed with BrdUrd for one hour or six hours after treatment with or without PSP (25 μg/ml) for 72 hours. Cells at G_0_/G_1 _and G_2_/M are BrdUrd negative labeled. Most of the S-phase cells actively undergoing DNA synthesis were labeled with BrdUrd. The one hour BrdUrd-labeled cells progressed through G_2_/M phase; divided after six hours of BrdUrd labeling and reappeared in the G_0_/G_1 _phase (6 hours BrdUrd labeled cells). These cells are called labeled divided cells (Ld) and the remaining S-phase cells are called labeled undivided cells (Lud). Values are expressed as mean (SD) (*n *= 5). * *P *< 0.05; ***P < 0.001, vs. control.

Bivariate BrdUrd/DNA analysis was used to investigate the effect of PSP on HL-60 cells. PSP induced accumulation of cells in the S-phase and G_2_/M phases by 56.1% (*P *< 0.001) and 44.9% (*P *< 0.001) respectively (Figure 4). The high level of BrdUrd-labeled cells in the S-phase suggests that PSP may cause some hyper-prolongation for DNA synthesis. DNA Ts increased from 9.76 hours to 11.70 hours (P = 0.045) in PSP-treated cells as measured by relative movement (Figure 5).

### Effect of CPT and PSP on the viability, necrosis and apoptosis HL-60 cells and PBMCs

**Figure 6:**
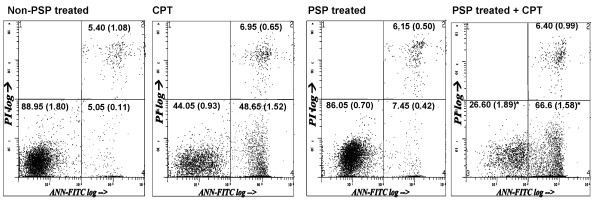
**Effect of PSP, CPT and PSP+CPT treatments on HL-60 apoptosis**. HL-60 cells were treated with PSP (25 μg/ml) for 72 hour before CPT (1 μM) was added. CPT was added at 72 hours and samples were collected after five hours of incubation. Cells were identified and quantified using annexin V/PI flow cytometric assay. R3 represents viable cells (PI-ve, annexin V-ve); R4 represents early apoptotic cells (PI-ve, annexin V+ve) and R2 represents late apoptotic cells (PI+ve, annexin V+ve). Values are expressed as means (SD) (*n *= 4). * *P *< 0.001, vs. CPT.

**Figure 7:**
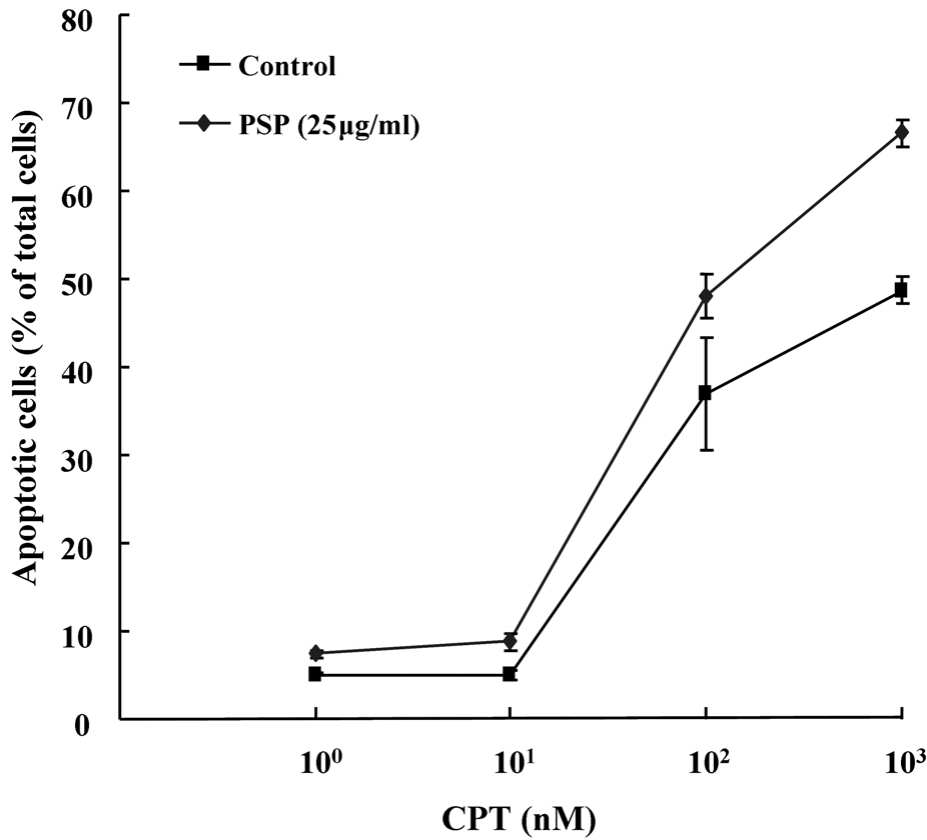
**Annexin V/PI flow cytometric analysis of apoptotic population of HL-60 cells treated with PSP, CPT and PSP+CPT treatments**. HL-60 cells were incubated with or without PSP (25 μg/ml) for 72 hours. The cells were subsequently exposed to CPT at concentrations of 0, 10, 100 and 1000 nM for five hours. Apoptotic cells were identified and quantified with flow cytometry with annexin V/PI staining. Results are expressed as means (SD) of four individual experiments analyzed with Winlist software.

With a S-phase synchronization strategy, we showed that pre-treatment of low dose PSP (25 μg/ml) for 72 hours was able to enhance the cytotoxicity of CPT (1 μM) to the HL-60 cells. The total viable, necrosis and apoptosis cells population were determined by annexin V/PI flow cytometry analysis in HL-60 cells and PBMCs in two separate experiments. CPT increased apoptosis from 5.05% to 48.65% (R4), whereas PSP treatment induced apoptosis (7.45%) in the HL-60 cells (Figure 6). Moreover, pre-treatment of HL-60 cells with PSP increased their sensitivity to CPT-induced apoptosis, raising the sensitive cell population from 48.65% (CPT alone) to 66.6% (PSP pre-treatment). We also tested further whether the apoptotic response of the PSP-treated HL-60 cells to CPT was linear with dose-dependent in a separate experiment. With doses of CPT ranged from 0, 10, 100 and 1000 nM for treatment of five hours, PSP pre-treated cells (25 μg/ml, 72 hours) were also enhanced the cytotoxicity of CPT (100 nM) by 30.12% (Figure 7). In contrast to the HL-60 cells, the PBMCs were not affected by treatment of PSP nor CPT alone, or with their combination (Table 1).

**Table 1:** The effect of CPT and CPT with PSP pretreatment on normal human peripheral blood mononuclear cells.

Treatment	Viable cells	Apoptotic cells	Necrotic cells
Control	37.93 (5.75)	29.62 (1.87)	31.51 (5.00)
PSP (25 μg/ml)	39.33 (4.70)	28.26 (2.18)	31.49 (4.11)
CPT (1 μM)	34.91 (5.39)	30.95 (2.49)	33.33 (5.05)
PSP (25 μg/ml) + CPT (1 μM)	40.80 (9.26)	28.85 (4.51)	29.38 (6.52)

Normal human peripheral blood mononuclear cells were treated with PSP (25 μg/ml) for 72 hour prior to addition of CPT (1 μM). CPT was added at 72 hours and samples were collected after five hours of drug incubation. Cells were identified and quantified using Annexin V/PI flow cytometric assay. Values are expressed as mean (SD) (*n *= 8).

### Change in cell cycle distribution of HL-60 cells with CPT treatment alone and with PSP pre-treatment

**Figure 8:**
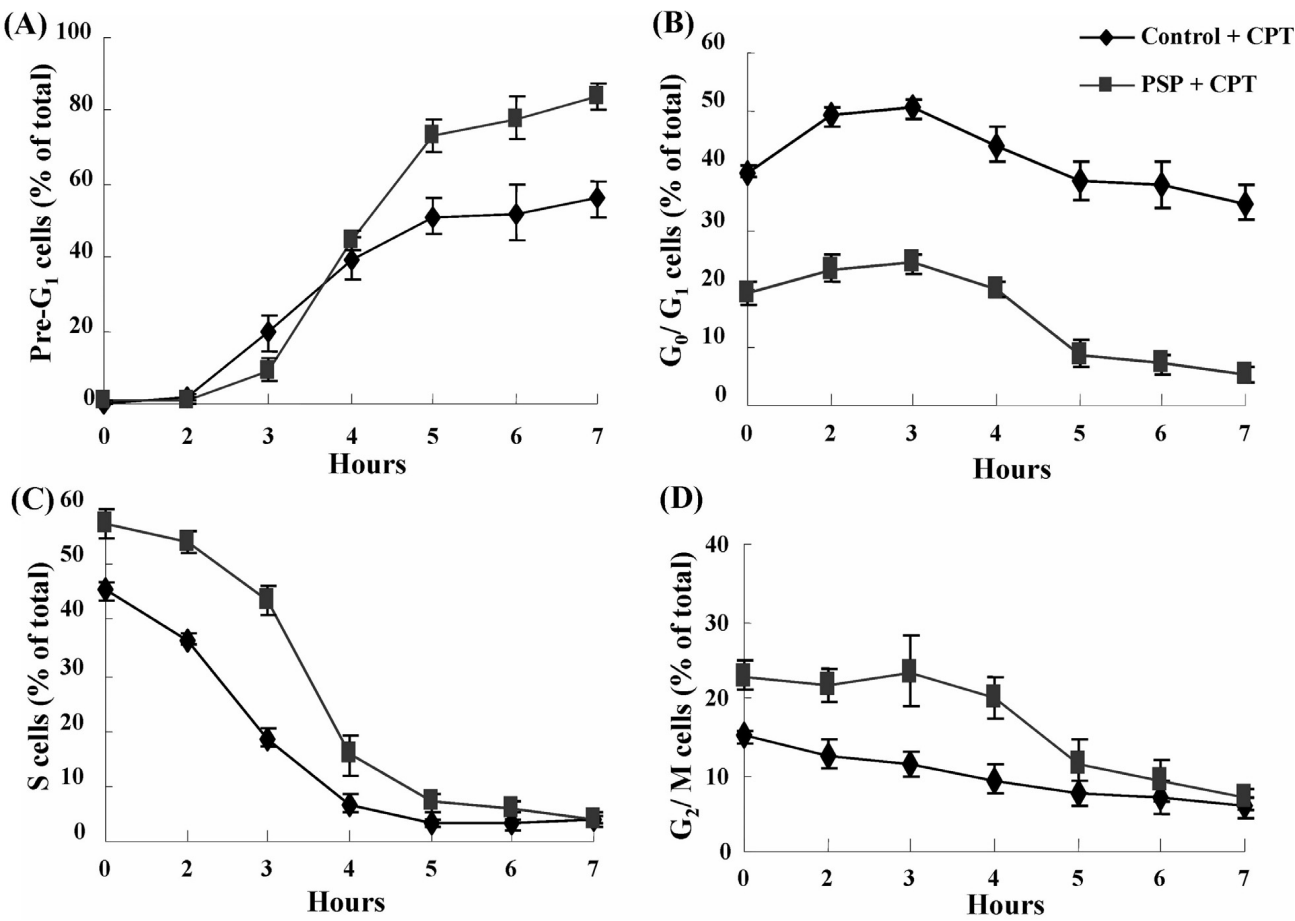
**DNA flow cytometric analysis of apoptotic population and changes in cell cycle distribution of HL-60 cells**. HL-60 cells were pre-treated with or without PSP (25 μg/ml) for 72 hours. The cells were subsequently exposed to CPT (1 μM) for up to seven hours. A time profile of the Pre-G_1 _peak (A) and the changes of cell cycle distribution in G_0_/G_1 _(B), S (C) and G_2_/M (D) were identified and quantified with flow cytometry with PI staining. Results are expressed as means (SD) of four individual experiments analyzed with Winlist and Modfit software.

Cell cycle distribution of HL-60 cells with or without PSP (25 μg/ml) pre-treatment differed after the addition of CPT (1 μM) (Figure 8). After incubation, CPT alone induced a 51.12% increase of the pre-G_1 _peak. Most of the S-phase cells (36.41%) were removed and only 14.71% of apoptotic cells were from the non-S-phase cells. On the other hand, combination treatment of PSP with CPT removed 50.42% of the S-phase cells. The remaining 22.74% of apoptotic cells were likely to be the non-S-phase cells.

### Effect of PSP, CPT and PSP pre-treatment with CPT on cyclin E and cyclin B_1 _expression in HL-60 cells

**Figure 9:**
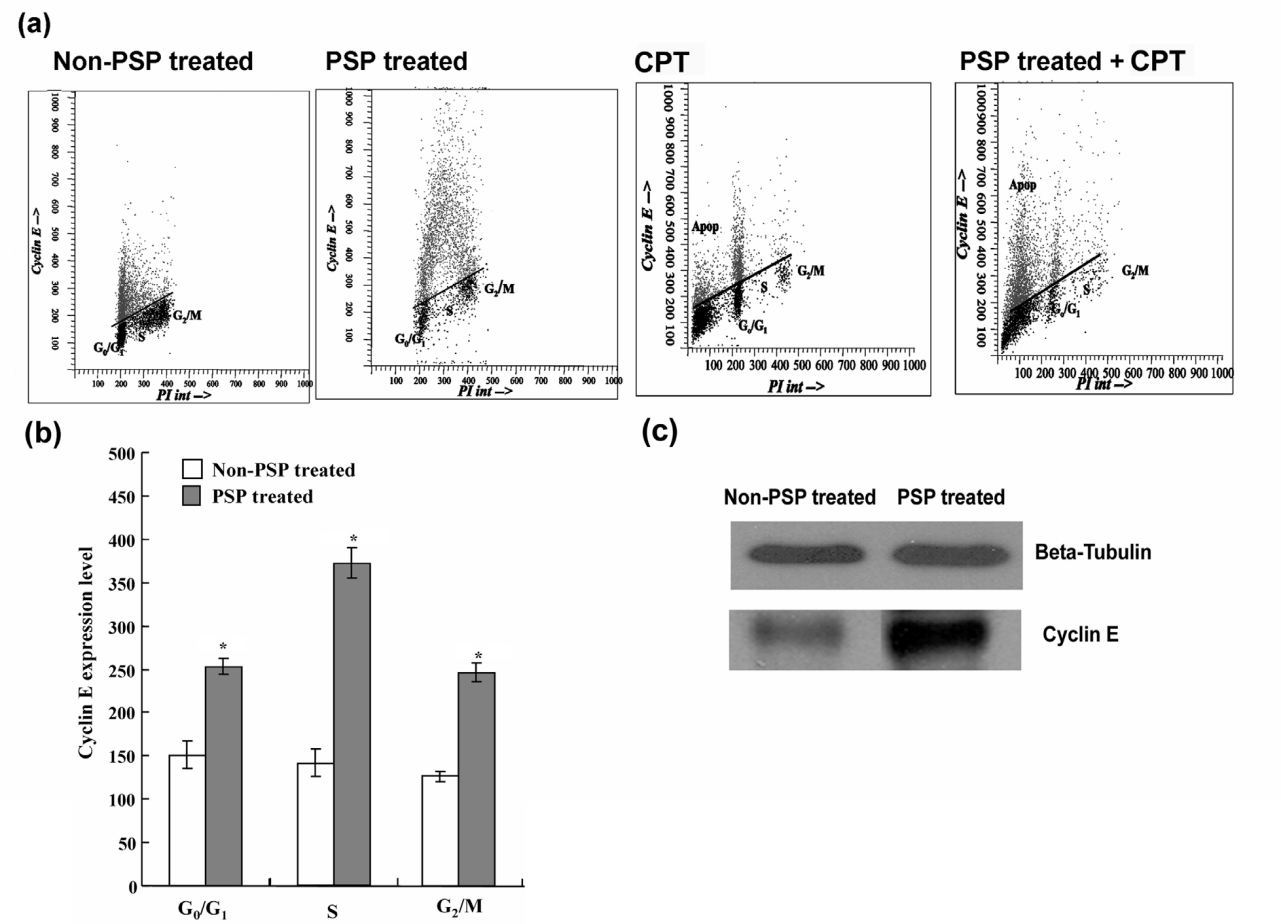
**Effect of PSP, CPT and PSP+CPT on cyclin E expression in HL-60 cells**. (a) HL-60 cells were treated or pre-treated with PSP (25 μg/ml) for 72 hour before CPT (1 μM) was added. CPT was added at 72 hours and samples were collected after five hours of incubation. (b) Cyclin E was expressed in non-PSP-treated cells (control) and PSP-treated HL-60 cells. Results are expressed as means (SD) of four individual experiments analyzed with Winlist and Modfit software. * *P *< 0.001, vs. non-PSP treated. (c) Western blot analysis of whole cell lysate of cyclin E expression in HL-60 cells with and without PSP treatment.

Cyclin E expression in the control HL-60 cells was the highest at G_0_/G_1_, slowly declined at late S-phase and was nearly undetectable at G_2_/M phase (Figure 9a). PSP treatment for 72 hours increased the cyclin E level in G_0_/G_1_, S and G_2_/M phases by 67.76% (*P *< 0.001), 163.46% (*P *< 0.001) and 93.91% (*P *< 0.001), respectively (Figure 9a and Figure 9b). Western blot analysis confirmed the up-regulation of cyclin E during PSP treatment (Figure 9c). After five hours of CPT (1 μM) incubation, the average cyclin E protein level in G_0_/G_1_, S and G_2_/M phase of the control (without PSP pre-treatment) was enhanced by 33.74%, 67.60% and 138.50% respectively (Figure 9a) while the average cyclin E protein level in the PSP pre-treatment group was not significantly changed. An increased proportion of pre-G_1 _cells expressing cyclin E suggested that cyclin E was involved in CPT-induced apoptosis in the S-phase cells.

**Figure 10:**
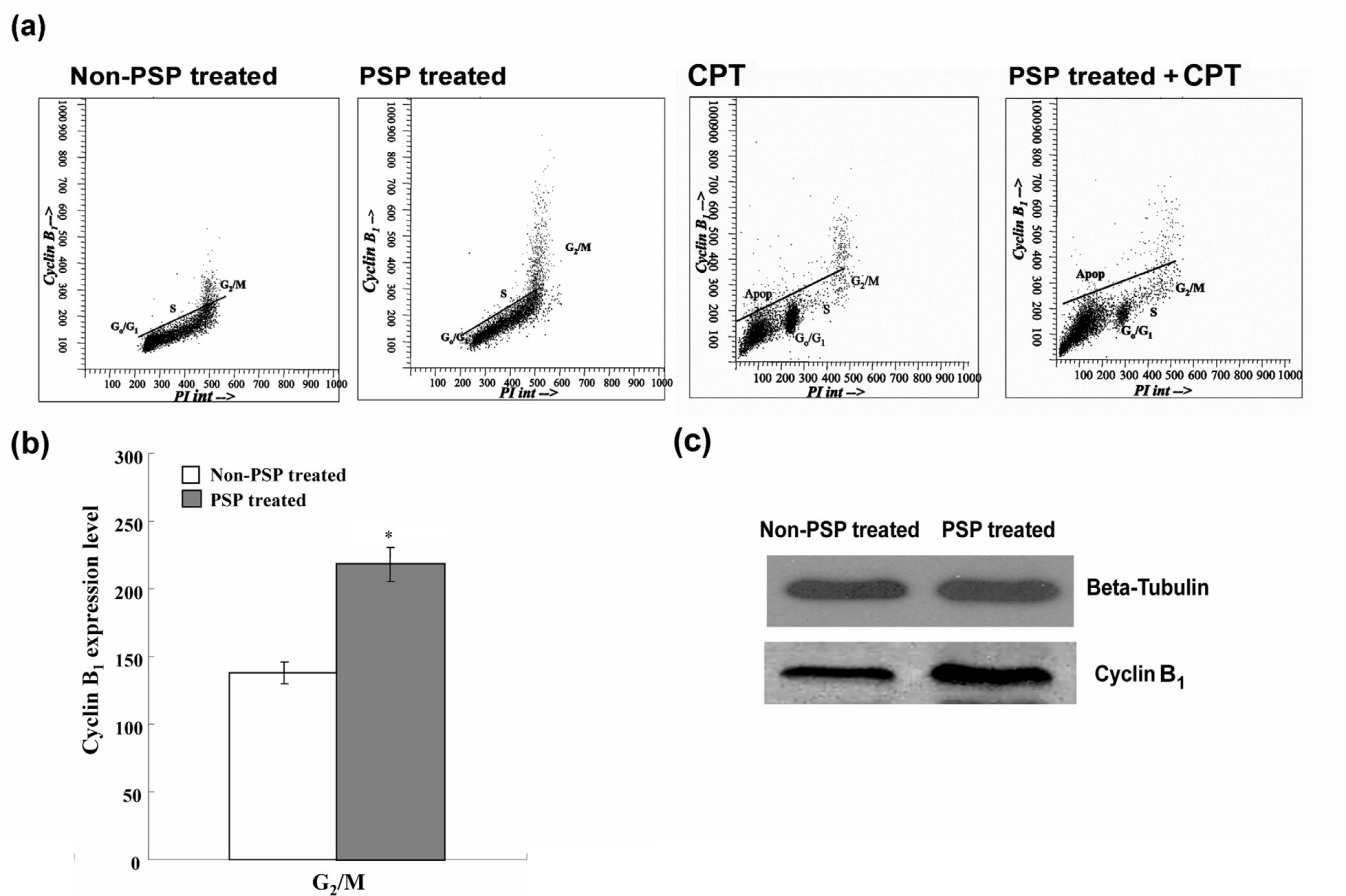
**Effect of PSP, CPT and PSP+CPT on cyclin B_1 _expression in HL-60 cells**. (a) HL-60 cells were pre-treated with PSP (25 μg/ml) for 72 hour before CPT (1 μM) was added. (b) Cyclin B_1 _was expressed in non-PSP-treated cells (control) and PSP-treated HL-60 cells. Results are expressed as means (SD) of four individual experiments analyzed with Winlist and Modfit software. * *P *< 0.001, vs. non-PSP treated. (c) Western blot analysis of whole cell lysate of cyclin B_1 _expression in HL-60 cells with and without PSP treatment.

The G_2_/M phase cells almost exclusively expressed cyclin B_1_(Figure 10a). PSP treatment for 72 hours increased the cyclin B_1 _level in G_2_/M phase by 58.44% (*P *< 0.001) (Figure 10b). Western blot analysis later confirmed the up-regulation of cyclin B_1 _during the PSP treatment (Figure 10c). Unlike cyclin E, the reduction of cyclin B_1 _was only specific to G_2_/M and to the non-proliferating cells. There was no cyclin B_1 _expression at the pre-G_1 _peak (i.e. apoptotic cells), indicating that most of the apoptotic cells in the pre-G_1 _peak came from the S-phase.

## Discussion

One of the advantages of PSP over many western anticancer drugs is its ability to distinguish cancer cells from the normal cells [4-6]. Moreover, PSP can synchronize cells and kill them in S-phase [5,6]. PSP is therefore a useful anticancer agent against rapidly proliferating tumor cells characterized by high proportion of S-phase cells exemplified by the HL-60 cells. The present study shows that PSP-pretreated cells enhanced the cytotoxicity of CPT.

Exposure of HL-60 cells to PSP delayed cell progression, predominantly through S and with lesser extent through G_2_. To a lesser degree, cells exiting G_2_/M were also impaired, significantly reducing G_1 _cells after PSP treatment. PSP increased the DNA content in HL-60 cells (detected with BrdUrd labeling); however, the rate of DNA synthesis was reduced (detected with [^3^H]-thymidine uptake).

CPT is cytotoxic to human promyelocytic HL-60 cells in both S and G_2 _phase [16]. Its increased toxicity towards S-phase cells may be explained by the possible collision between moving replication forks and CPT-stabilized topoisomerase I-DNA cleavable complexes, resulting in fork arrest and its breakage [17]. We previously reported that PSP inhibited topoisomerase IIα in the HL-60 cells [5]. It is not clear whether PSP exhibits similar effect as CPT in terms of inhibiting the TOP1 activity. Further studies are warranted to test such a possibility.

Similar to many anticancer agents, PSP is capable of inducing DNA fragmentation (pre-G_1 _peak) at higher doses (100 μg/ml) [5]. The present study demonstrated that PSP enhanced cytotoxicity or the sensitivity of the HL-60 cells induced by CPT (detected with annexin V/PI flow cytometry). Flow cytometry analysis showed that PSP enhanced the anticancer effects of CPT via either cell cycle perturbation at low dose or apoptotic machinery at high dose. PSP at low dose (25 μg/ml) caused S-phase accumulation, which may be up-regulated by the G_1_/S checkpoint cyclin E. The pre-G_1 _population increased with a significant decrease in S-phase population, suggesting that PSP must first accumulate the S-phase cells before exerting its cytotoxic effect on the HL-60 cells. Our propose that the ability of PSP to arrest cancer cells in the DNA synthesis (S) phase PSP sensitizes the HL-60 cells to undergo apoptosis induced by CPT.

Study results indicated that cyclin E was more important than cyclin B_1 _in CPT-induced apoptosis in the HL-60 cells. Cyclin E is essential in the cell cycle from G_1 _to S-phase and the initiation of DNA replication [18-21]. PSP-induced cyclin E expression led to S-phase cell accumulation, which subsequently delayed DNA synthesis, hence a longer Ts in the PSP pre-treated HL-60 cells. In addition to the regulatory role in G_1_-S transition, cyclin E may also be involved in apoptosis. A recent study demonstrated that the induction of cyclin E expression sensitized the IM-9 cells to γ-irradiation-induced apoptosis [22]. Irradiation-induced cyclin E overexpression could result in an enhanced caspases activation and phosphatidylserine translocation on the plasma membrane, leading to cell death in the IM-9 cells [22]. Furthermore, DNA-damage-induced cyclin E-H1K was suggested to be involved in a late apoptosis checkpoint [23]. We believe that the up-regulation of the cyclin E may be a sensitization process of PSP whereby the HL-60 cells undergo DNA damage by CPT. Consequently, a larger number of protein-associated DNA strand breaks may lead to more pronounced apoptosis induction.

## Conclusion

The present study shows that PSP extracted from *Coriolus versicolor *can increase the sensitization of the HL-60 cells to undergo effective apoptotic cell death induced by CPT. The pattern of sensitivity of cancer cells is similar to that of HL-60 cells. PSP rapidly arrests and/or kills cells in S-phase and did not interfere with the anticancer action of CPT. PSP is a potential adjuvant to treat human leukemia as rapidly proliferating tumors is characterized by a high proportion of S-phase cells.

## Abbreviations

PSP: polysaccharopeptide; CPT: Camptothecin; HL-60: human promyelocytic leukemia cell line; BrdUrd: bromodeoxyuridine; TOP1: topoisomerase I; RM: relative movement; Ts: DNA synthesis time; DTT: 1, 4-Dithiothreitol; PBS: phosphate buffer saline; PI: propidium iodide; PBMCs: Human peripheral blood mononuclear cells; SD: standard deviation.

## Competing interests

The authors declare that they have no competing interests.

## Authors' contributions

JMFW designed the study, interpreted the data and drafted the manuscript. WHS, XTY, PPJ and LLYW conducted the experiments and analyzed the data. All authors read and approved the final version of the manuscript.
